# Mediating role of family and workplace support on health-related quality of life among self-reported young adult cancer survivors: a cross-sectional observational study

**DOI:** 10.1186/s41687-026-01120-2

**Published:** 2026-06-11

**Authors:** Takafumi Soejima, Mari Kitao

**Affiliations:** Graduate School of Medicine, Kobe University, 7-10-2 Tomogaoka, Suma-ku, Hyogo Japan

**Keywords:** Cancer survivors, Long term adverse effects, Quality of life, Social support, Young adult

## Abstract

**Background:**

The influence of family and workplace support on different domains of health-related quality of life (HRQOL) remains unclear in optimizing psychosocial interventions to improve HRQOL for young adult cancer survivors. In this study, we aimed to clarify the mediating effects of family and workplace support on the association between cancer-related symptoms and each domain of HRQOL among young adults with a self-reported cancer diagnosis (self-reported young adult cancer survivors).

**Methodology:**

This cross-sectional observational study used an online survey conducted in Japan in January 2022. The survey was designed to assess demographic and clinical characteristics, physical fatigue (using Physical Fatigue subscale of the Cancer Fatigue Scale), depression (using Kessler-6), cognitive impairments (using Cognitive Functioning subscale of the European Organization for Research and Treatment of Cancer Quality of Life Questionnaire Core 30), family support (using a subscale of the Multidimensional Scale of Perceived Social Support), workplace support (using a subscale of the New Brief Job Stress Questionnaire), and HRQOL (using the 12-Item Short-Form Health Survey). We conducted structural equation modeling (SEM) using data from 192 participants.

**Results:**

Of the 192 participants, 78% were female, 60% had undergone surgery, and 67% were permanent workers. Notably, the mean scores in the social domain of HRQOL were significantly lower than the national standard values in the Japanese population aged 20–39 years. The indirect effects of workplace support were significant for the association of cancer-related symptoms with mental (β = -0.07) and social (β = -0.06) domains of HRQOL. Similarly, the indirect effects of family support were significant for the association of cancer-related symptoms with physical (β = -0.06) and social (β = -0.05) domains of HRQOL.

**Conclusions:**

Family support mediated the association of cancer-related symptoms with physical and social QOL, whereas workplace support mediated the association of cancer-related symptoms with mental and social QOL. The social QOL among self-reported young adult cancer survivors was lower than that of the general Japanese population aged 20–39 years. Therefore, strategies targeting family and workplace support may be crucial for Japanese young adult cancer survivors in maintaining and improving their social QOL.

## Abstract (plain language summary)

Our study clarified family and workplace support related to the physical, psychological, and social aspects of health-related quality of life among self-reported young adult cancer survivors. These findings suggest more optimized strategies to improve their health-related quality of life.

## Background

Ongoing progress in cancer treatment has led to sustained increases in the 5-year survival rate for young adult cancer survivors [1, 2]. Consequently, the number of young adult cancer survivors, typically defined as individuals aged 20–39 diagnosed with adult-onset cancer, is projected to increase [3]. Following treatment, young adult cancer survivors experience various physical (lack of energy, feeling drowsy, sleep disturbance, and pain), psychological (depression and anxiety), and cognitive problems (difficulties remembering things and paying attention) [4–6]. This occurs during a critical period when they are typically completing their education, starting their employment pathways, and achieving social and financial independence from their parents [7, 8]. However, young adult cancer survivors reported lower vocational and educational attainment [9, 10] and greater financial burden [11] due to the persistent physical, mental, and cognitive symptoms of the disease. Consequently, young adult cancer survivors have lower health-related quality of life (HRQOL) than the general population or individuals without a history of cancer, even long after treatment completing [12, 13].

Cancer and its treatment have various adverse effects on survivors, prompting the development of coping skills and social support for self-defense [14, 15]. Previous studies indicated a relationship of physical (difficulties in performing daily activities) and mental health problems (depression and anxiety) with social support among cancer survivors [16, 17]. Furthermore, acquiring coping skills and social support as a protective response improves well-being in cancer survivors [14, 15]. Previous studies suggested that disease- and treatment-related physical and psychological problems influence HRQOL through social support among individuals with chronic illnesses including cancer [18–20]. Despite the significant impact of social support on overall HRQOL among cancer survivors, few studies have individually explored its association with multiple HRQOL domains [21, 22]. Therefore, social support may be differentially associated with the physical, mental, and social domains of young adult cancer survivors’ HRQOL.

Among young adult cancer survivors, family social support is particularly vital for maintaining and improving HRQOL [23, 24]. Among cancer survivors, family support could help with activities of daily living including assistance with household chores, empower cancer survivors to make decisions about healthcare providers or treatment, and enhance feelings of well-being by having family members to talk to [25, 26]. Thus, family support may bolster the physical and mental well-being of young adult cancer survivors who navigated the survivorship trajectory. Additionally, young adult cancer survivors are in their initial stages of career development [27]. Workplace support and job accommodations positively impacts return-to-work experience and job performance among cancer survivors experiencing cancer-related complications, which can lead to employment continuation [28–30]. Pursuing employment has been shown to reduce financial burden, enhance self-esteem, and foster a sense of normalcy, thereby improving health-related quality of life among cancer survivors [31–33]. Workplace support could stabilize the young adult cancer survivors’ working life and improve their psychological aspects including self-efficacy and a sense of normalcy, which may enhance their mantal and social QOL. However, it remains unclear how family and workplace support influence different aspects of HRQOL when optimizing psychosocial interventions to improve HRQOL among young adult cancer survivors.

In this study, we aimed (1) to clarify the differences in HRQOL between young adults with a self-reported cancer diagnosis (self-reported young adult cancer survivors) and Japanese general population and (2) to identify the mediating effects of family and workplace support on the relationship between cancer-related symptoms (physical fatigue, depression, and cognitive impairment) and HRQOL (physical, mental, and social QOL) among self-reported young adult cancer survivors. We hypothesized (1) that self-reported young adult cancer survivors could report lower physical, mental, and social QOL than Japanese general population, and (2) that family support could mediate the association of cancer-related symptoms with physical and mental QOL, and workplace support could mediate the association of cancer-related symptoms with mental and social QOL. Identifying this mediating effect would help suggest and devise effective family or workplace support interventions to improve young adult cancer survivors’ HRQOL, considering the inhibited aspects of their HRQOL.

## Methods

### Study participants and procedure

This cross-sectional observational study was part of a research project investigating work sustainability among young adult cancer survivors, and the participant recruitment process has been previously reported [34]. In January 2022, we conducted a web-based survey of self-reported cancer survivors aged 20–39 years registered with a commercial panel system provided by Macromill, Inc. (https://group.macromill.com), an Internet research company in Japan. First, this study screened potential participants who registered with the commercial panel system, gathering information on cancer sites and treatment. From the commercial panel system, 6,161 participants between 20 and 39 years old who were diagnosed with cancer were identified by this screening and were sent online questionnaires. Second, we again asked our study participants for the same questions on cancer sites and treatment as the screening in the questionnaires. Participants whose responses in the questionnaires differed from answers at the screening were excluded. We finally included data from participants who met the following criteria: (a) aged 20–39 years at the time of the survey, (b) diagnosed with cancer at age 20 years or older, (c) completed cancer treatment, (d) employed at the time of the survey, and (e) understood the purpose of this study and completed a questionnaire in Japanese.

The sample size was calculated using the package of “semTools” of R version 4.5.0 under the condition where the probability of judging a model to be a very poor fit (root mean square error of approximation, RMSEA = 0.1) but not is 0.05, the probability of judging a model to be a very good fit (RMSEA = 0.01) when it was determined that it was not a very poor fit is 0.8, and degrees of freedom is 11. As a result, data from 166 or more participants were required in this study.

### Measures

Participants provided the following demographic and clinical characteristics through a questionnaire: age at the time of the survey, sex, educational level, marital status, employment status, occupation, cancer site, period from diagnosis, treatment, metastasis, relapse, and job demands. The New Brief Job Stress Questionnaire (New BJSQ) to assess job demands [35]. Subscale scores were calculated as the sum of the item scores divided by the number of items. The scores were not calculated if more than half the items had missing responses,. Subscale scores ranged from 1 to 4, with higher scores indicating greater job demands. Cronbach’s alpha in this study was 0.87.

The CFS Physical Fatigue subscale was used to measure the participants’ physical fatigue [36]. This seven-scale consists uses a five-point Likert scale. The subscale score was calculated as the sum of the scores of all items. The score was not calculated if more than half the items had missing responses. The score ranges from 0 to 28 for this subscale, with higher score indicating more severe physical fatigue. Cronbach’s alpha in this study was 0.94.

Depressive symptoms were measure using the K6 among the participants [37], a six items using a five-point Likert scale. The K6 score is calculated as the sum of the scores of all items. The K6 score was not calculated if more than half of the items had missing responses. The scores ranged from 0 to 24, with higher scores indicating more severe depressive symptoms. Cronbach’s alpha in this study was 0.94.

Participants’ cognitive impairment was assessed using the Cognitive Functioning subscale of the EORTC QLQ-C30 [38]. This two-item subscale uses a four-point Likert scale. Subscale scores were calculated by transforming the average score for each item from to 0–100. If all items had missing responses, a score was not calculated. Higher scores indicate more severe cognitive impairment. This subscale has been used in numerous studies to assess cognitive impairment in cancer survivors [39]. Cronbach’s alpha in this study was 0.80.

Family Support was assessed using a four-item subscale of the Multidimensional Scale of Perceived Social Support (MSPSS) [40], rated using a seven-point Likert scale. Subscale scores were calculated as the sum of the item scores divided by the number of items. The score was not calculated if more than half of the items had missing responses. The possible subscale scores response range was 1–7, with higher scores indicating greater family support. The validity and reliability of the Japanese version of the MSPSS, including internal consistency, factorial validity, concurrent validity against satisfaction with parent-child and marital relationships, and known-group validity concerning mental problems and marital status, were confirmed among Japanese middle-aged and older adults [40]. Cronbach’s alpha in this study was 0.95.

Support from coworkers and supervisors was assessed using the New BJSQ subscales [35]. Subscale scores were calculated as the sum of the item scores divided by the number of items. The score was not calculated if more than half of the items had missing responses. The possible response range for the subscale scores was 1–4, with higher scores indicating more coworker and supervisor support. The Cronbach’s alpha in this study was 0.87 and 0.89 for the subscale on support from coworkers and supervisors, respectively.

HRQOL was assessed using the 12-Item Short-Form Health Survey (SF-12) of the participants [41]. This 12-item instrument uses three-to six-category Likert response scales to generate three summary scores: Physical Component Summary (PCS), Mental Component Summary (MCS), and Role/Social Component Summary (RCS). Each summary score was weighted using a norm-based scoring method. Higher PCS, MCS, and RCS scores indicated better physical, mental, and social QOL, respectively. Cronbach’s alpha for the SF-12 total in this study was 0.86.

### Statistical analysis

All analyses were performed using IBM SPSS and Amos software version 28 (SPSS Inc., Chicago, IL, USA). We calculated the frequencies, percentages, means, and standard deviations (SD) of demographic and clinical characteristics, cancer-related symptoms, family and workplace support, and HRQOL. Spearman’s rank correlation analysis was used to identify demographic and clinical characteristics that potentially confounded the relationships between cancer-related symptoms, family and workplace support, and HRQOL. The Spearman’s rank correlation coefficients were significant when the p-values for these coefficients were under 0.05 (two-tailed). The participants’ scores of PCS, MCS, and RCS of SF12 were compared with these national standard values in the respective Japanese population age groups [41] using Welch’s test. When the p-value were below 0.05 (two-tailed) based on the Welch’s test, we judged that the participants’ PCS, MCS, and RCS scores on the SF-12 were different from these national standard values. Furthermore, Cohen’s d (≥ 0.2, small; 0.5, medium; 0.8, large) were calculated as effect sizes [42].

Structural equation modeling (SEM) was performed using a maximum likelihood method to clarify the mediating effect of the association between cancer-related symptoms and HRQOL among the participants. The SEM model included the main variables (cancer-related symptoms, family and workplace support, and HRQOL) and any demographic or clinical variable that significantly correlated with any of the PCS, MCS, and RCS of SF-12 in the Spearman’s rank correlation analysis as adjusted variables. We set in the SEM model not only the paths among the main variables but also the paths from the adjusted variables to the PCS, MCS, and RCS, and retained the significant covariances between adjusted variables and the main variables. Acceptance fit of the SEM model is indicated by chi-square statistics divided by degrees of freedom (CMIN/df) < 3, comparative fit index (CFI) > 0.95, goodness-of-fit index (GFI) > 0.90, adjusted goodness-of-fit index (AGFI) > 0.85, and RMSEA < 0.08 [43]. Standardized path coefficients (β) between variables and for mediating effects with 95% confidence intervals (CIs) were estimated using a bootstrap procedure with 2,000 resamples. The significance of βs between variables and for mediating effects was tested using the bias-corrected bootstrap test. If the p-values for βs were below 0.05 (two-tailed) based on a bootstrap analysis, the associations between variables were statistically significant. The βs are 0.1 for a small effect size, 0.3 for a moderate effect size, and 0.5 for a large effect size, and indirect effects are 0.01 for a small effect size, 0.09 for a moderate effect size, and 0.25 for a large effect size [42].

### Ethical consideration

Ethical approval for this study was obtained from the Ethics Committee of the principal investigator’s institution (August 31, 2021; No. 1029), and the research was carried out in accordance with the Declaration of Helsinki and the Ethical Guidelines for Medical and Biological Research Involving Human Subjects established by the Ministry of Health, Labor, and Welfare.

## Results

### Participants characteristics

Of the 6,161 participants, 693 returned the online questionnaires (response rate was 11.2%). One hundred thirteen of 693 participants whose responses on cancer sites and treatment in the questionnaires differed from answers at the screening were excluded. We excluded participants who: were diagnosed with cancer before age 20 (*n* = 138); did not complete cancer treatment (*n* = 153); were not working at the time of the survey (*n* = 70); and did not complete the SF-12 (*n* = 27). Finally, we analyzed data from 192 participants.

Of the 192 participants, 52% were aged 35–39 years, 78% were female, 42% were diagnosed with uterine and ovarian cancer, 37% were diagnosed at 5 years or older, and 60% underwent surgery (Table 1). A longer period from diagnosis was positively correlated with PCS (*r* = 0.19, *p* = 0.010), MCS (*r* = 0.19, *p* = 0.009), and RCS (*r* = 0.30, *p* < 0.001). Undergoing surgery was positively correlated with PCS (*r* = 0.19, *p* = 0.009) and MCS (*r* = 0.15, *p* = 0.045).

**Table 1:** Demographic and clinical characteristics of participants (*N* = 192)

			PCS		MCS		RCS	
	*n*	%	*r* ^a^	*p* ^a^	*r* ^a^	*p* ^a^	*r* ^a^	*p* ^a^
Age at the Time of the Survey			0.07	0.332	-0.01	0.940	0.08	0.252
20–24 years	8	4.2						
25–29 years	24	12.5						
30–34 years	60	31.3						
35–39 years	100	52.1						
Sex ^b^			0.02	0.767	-0.10	0.191	0.14	0.063
Male	43	22.4						
Female	149	77.6						
Educational Level ^c^			0.07	0.306	-0.03	0.698	-0.10	0.168
High School Graduate or Less	43	22.4						
More than High School Graduate	146	76.0						
Others	3	1.6						
Marital Status ^d^			-0.01	0.913	0.08	0.251	-0.07	0.328
Married	82	42.7						
Not Married	110	57.3						
Employment Status ^e^			0.03	0.730	0.02	0.787	0.03	0.735
Permanent	128	66.7						
Temporary	55	28.6						
Self-employed	9	4.7						
Occupation ^f^			0.14	0.062	-0.09	0.209	0.06	0.416
Technical	37	19.3						
Managerial	17	8.9						
Clerical	70	36.5						
Sales	20	10.4						
Production	16	8.3						
Services	23	12.0						
Others	9	4.7						
Cancer Sites ^g^			0.09	0.223	-0.08	0.270	0.10	0.171
Gastric Cancer	15	7.8						
Colorectal Cancer	13	6.8						
Breast Cancer	17	8.9						
Uterine and Ovarian Cancer	80	41.7						
Thyroid cancer	17	8.9						
Lymphoma/Leukemia	15	7.8						
Others	35	18.2						
Period from Diagnosis			0.19	0.010	0.19	0.009	0.30	< 0.001
< 1 year	31	16.1						
1- < 5 years	91	47.4						
≥ 5 years	70	36.5						
Treatment ^h^								
Chemotherapy	48	25.0	-0.13	0.084	0.13	0.073	-0.10	0.155
Surgery	115	59.9	0.19	0.009	0.15	0.045	0.13	0.067
Radiation	25	13.0	-0.02	0.741	0.06	0.395	-0.06	0.439
Hormone therapy	17	8.9	0.03	0.657	0.01	0.864	-0.03	0.652
Metastasis and/or Relapse	10	5.2	0.01	0.931	0.02	0.742	0.04	0.600
	Mean	SD	r ^a^	p ^a^	r ^a^	p ^a^	r ^a^	p ^a^
Job demands (New BJSQ)	1.6	0.7	0.03	0.661	-0.14	0.063	-0.12	0.101
Support from Coworker (New BJSQ)	1.4	0.8	0.06	0.415	0.31	< 0.001	0.10	0.182
Support from Supervisor (New BJSQ)	1.3	0.8	-0.03	0.728	0.42	< 0.001	0.13	0.084
Family Support (MSPSS)	4.3	1.6	0.25	< 0.001	0.01	0.863	0.33	< 0.001
Physical Fatigue (CFS)	8.9	7.4	-0.21	0.003	-0.41	< 0.001	-0.32	< 0.001
Depression (K6)	6.3	6.3	-0.17	0.020	-0.38	< 0.001	-0.44	< 0.001
Cognitive Impairment (EORTC QLQ-C30)	68.0	26.6	0.18	0.012	0.19	0.008	0.34	< 0.001

Note. CFS, Cancer Fatigue Scale; EORTC QLQ-C30, European Organization for Research and Treatment of Cancer Quality of Life Questionnaire Core 30; K6, Kessler-6; MCS, Mental Component Summary; MSPSS, Multidimensional Scale of Perceived Social Support; New BJSQ, New Brief Job Stress Questionnaire; PCS, Physical Component Summary; RCS, Role/social Component Summary; SD, standard deviation. ^a^ The Spearman’s ranked order correlation coefficients between characteristics and each summary score of SF-12, and p-values were calculated. ^b^ The correlation coefficient was calculated using dichotomous variables (Female = 1, Male = 0). ^c^ The correlation coefficient was calculated using dichotomous variables (College, Vocational School, University, Graduate school = 1, Others = 0). ^d^ The correlation coefficient was calculated using dichotomous variables (Married = 1, Not married = 0). ^e^ The correlation coefficient was calculated using the dichotomous variables (Permanent = 1, Temporary and self-employed = 0). ^f^ The correlation coefficient was calculated using the dichotomous variables (Clerical = 1, Others = 0). ^g^ The correlation coefficient was calculated using the dichotomous variables (Female Genital Cancer = 1, Others = 0). ^h^ Multiple answers allowed

### Participants’ score of HRQOL

The mean scores of PCS, MCS, and RCS on SF-12 for the total participants were 49.9 (SD 12.1), 49.6 (SD 10.6), and 40.4 (SD 14.9), respectively (Table 2). The mean scores of PCS and RCS in both participants aged 20–29 (mean 48.6, SD 12.2 in PCS and mean 35.7, SD 14.2 in RCS) and 30–39 years (mean 50.1, SD 12.1 in PCS and mean 41.3, SD 14.9 in RCS), were significantly lower than the national standard value of PCS and RCS in the respective Japanese population age groups. The effect sizes for differences in RCS between the participants and the Japanese population were especially medium or large (d = 1.01 for ages 20–29 and d = 0.78 for ages 30–39).

**Table 2:** PCS, MCS, and RCS Scores of SF-12 in participants (*N* = 192)

	Study Participants			Japanese General Population					
	*n*	Mean	SD	*n*	Mean	SD	t ^a^	*p* ^a^	d ^b^
Total									
PCS	192	49.9	12.1						
MCS	192	49.6	10.6						
RCS	192	40.4	14.9						
20–29 years									
PCS	32	48.6	12.2	269	55.2	8.2	-2.98	0.005	0.76
MCS	32	49.3	11.9	269	49.8	10.1	-0.23	0.821	0.05
RCS	32	35.7	14.2	269	47.5	11.3	-4.53	< 0.001	1.01
30–39 years									
PCS	160	50.1	12.1	394	52.7	8.3	-2.48	0.014	0.27
MCS	160	49.7	10.4	394	49.6	9.2	0.11	0.911	0.01
RCS	160	41.3	14.9	394	50.0	9.2	-6.83	< 0.001	0.78

Note. MCS, Mental Component Summary; PCS, Physical Component Summary; RCS, Role/Social Component Summary. ^a^ The differences between the scores of the current study and national standard values in Japanese population was tested using Welch’s test. ^b^ Cohen’s d were calculated

### Mediating role of workplace and family support on HRQOL

The SEM model in which the dependent variables were PCS and MCS included the variables on period from diagnosis and receiving surgery which were significantly correlated with PCS and MCS in the bivariate analyses. The SEM model in which the dependent variables were RCS included only the variable on period from diagnosis which were significantly correlated with RCS in the bivariate analyses. The SEM models including the main variables (cancer-related symptoms, workplace and family support, and HRQOL) along with period from diagnosis and/or receiving surgery (variables significantly correlated with HRQOL in the bivariate analyses) were confirmed using the indicators of model fit (Table 3). The models in which the dependent variables were PCS, MCS, or RCS demonstrated acceptance fit to the data in the basis of indices of CMIN/df, CFI, GFI, AGFI, and RMSEA. Thus, these SEM models were used for further analysis.

**Table 3:** Model Fit in Models (*N* = 192)

Dependent variables	CMIN/df	CFI	GFI	AGFI	RMSEA
PCS	1.613	0.978	0.966	0.924	0.057
MCS	1.769	0.975	0.962	0.915	0.063
RCS	1.718	0.982	0.971	0.930	0.061

Note. AGFI, Adjusted Goodness-of-Fit Index; CFI, Comparative Fit Index; CMIN/df, Chi-square statistics divided by degrees of freedom; GFI, Goodness-of-Fit Index; RMSEA, Root Mean Square Error of Approximation. The model included the main variables (cancer-related symptoms, workplace and family support, and HRQOL) along with time since diagnosis and receiving surgery (variables significantly correlated with HRQOL in the bivariate analyses)

**Fig. 1:**
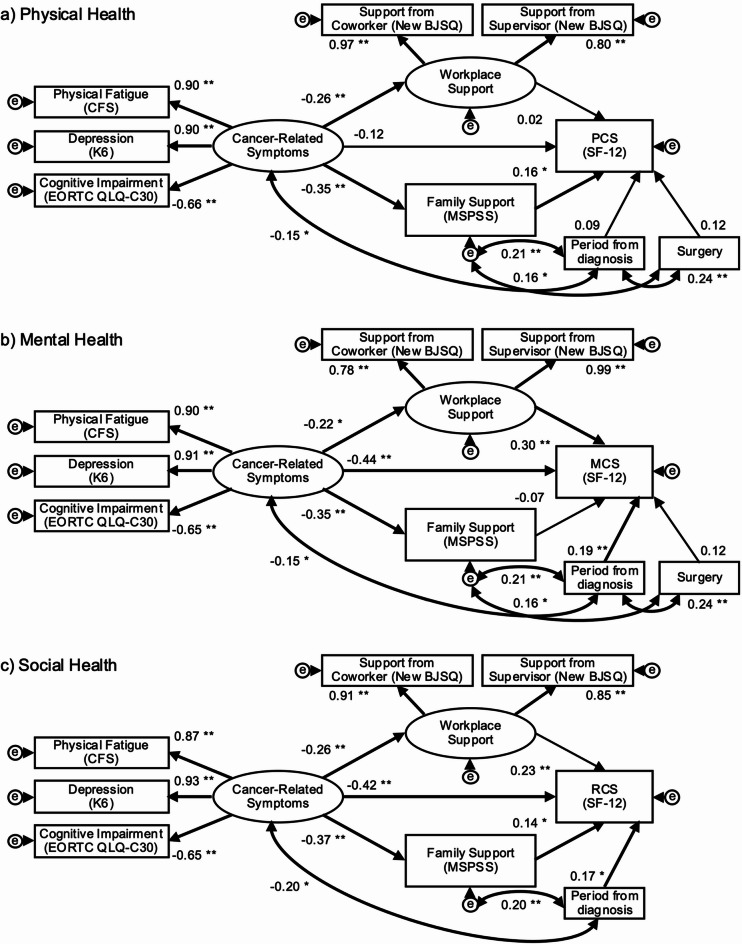
Model of the mediating role of family and workplace support on HRQOL: Note. CFS, Cancer Fatigue Scale; EORTC QLQ-C30, European Organization for Research and Treatment of Cancer Quality of Life Questionnaire Core 30; K6, Kessler-6; MCS, Mental Component Summary; MSPSS, Multidimensional Scale of Perceived Social Support; New BJSQ, New Brief Job Stress Questionnaire; PCS, Physical Component Summary; RCS, Role/social Component Summary; SD, standard deviation. The results of models including (**a**) PCS, (**b**) MCS, and (**c**) RCS scores of SF-12 as dependent variables are shown. The path coefficients are shown as numerical values alongside the respective arrows. The latent variable “e” implies the error of each variable. The significant paths are indicated in bold (* *p* < 0.05; ** *p* < 0.01)

In the model with PCS as the dependent variables, cancer-related symptoms reduced family (β = -0.35) and workplace support (β = -0.26), while family support improved PCS (β = 0.16), which indicated effect sizes ranging between small and moderate (Fig. 1; Table 4). In the model including MCS as the dependent variables, cancer-related symptoms reduced family (β = -0.35) and workplace support (β = -0.22), while workplace support improved MCS (β = 0.30), which indicated effect sizes ranging between small and moderate. In the model including RCS as the dependent variables, cancer-related symptoms reduced family (β = -0.37) and workplace support (β = -0.26), and both family (β = 0.14) and workplace support (β = 0.23) improved RCS, which indicated effect sizes ranging between small and moderate. Family support mediated the association of cancer-related symptoms with PCS (β = -0.06) and RCS (β = -0.05) with small effect sizes. Additionally, workplace support mediated the association of cancer-related symptoms with MCS (β = -0.07) and RCS (β = -0.06) with small effect sizes.

**Table 4:** Standardized path coefficients between cancer-related symptoms, family and workplace support, and HRQOL (*N* = 192)

	Dependent Variables											
	PCS				MCS				RCS			
	95%CI				95%CI				95%CI			
	β	Upper	Lower	p	β	Upper	Lower	p	β	Upper	Lower	p
Direct Effects												
Cancer-Related Symptoms												
→ Family Support	-0.35	-0.47	-0.21	0.001	-0.35	-0.47	-0.20	0.001	-0.37	-0.49	-0.22	0.001
→ Workplace Support	-0.26	-0.41	-0.08	0.005	-0.22	-0.39	-0.02	0.017	-0.26	-0.42	-0.10	0.002
→ PCS	-0.12	-0.28	0.07	0.207								
→ MCS					-0.44	-0.56	-0.28	0.002				
→ RCS									-0.42	-0.57	-0.28	0.001
Family Support												
→ PCS	0.16	0.01	0.31	0.041								
→ MCS					-0.07	-0.20	0.06	0.300				
→ RCS									0.14	0.001	0.27	0.049
Workplace Support												
→ PCS	0.02	-0.17	0.16	0.848								
→ MCS					0.30	0.17	0.43	0.003				
→ RCS									0.23	0.08	0.36	0.002
Indirect Effects												
Cancer-Related Symptoms												
→ Family Support → PCS	-0.06	-0.12	-0.004	0.030								
→ Family Support → MCS					0.03	-0.019	0.082	0.254				
→ Family Support → RCS									-0.05	-0.12	-0.003	0.042
→ Workplace Support → PCS	-0.004	-0.05	0.05	0.801								
→ Workplace Support → MCS					-0.07	-0.14	-0.01	0.011				
→ Workplace Support → RCS									-0.06	-0.13	-0.02	0.002

Note. β, Standardized Path Coefficients; CI, Confidence Interval; HRQOL, Health-Related Quality of Life; MCS, Mental Component Summary; PCS, Physical Component Summary; RCS, Role/social Component Summary

## Discussion

In our study, self-reported young adult cancer survivors had lower physical and social QOL than the general Japanese population aged 20–39 years. Furthermore, our findings suggest that family support fully mediates the relationship between cancer-related symptoms and physical QOL, workplace support partially mediates the relationship between cancer-related symptoms and mental QOL, and both family and workplace support partially mediate the relationship between cancer-related symptoms and social QOL. Previous studies have shown an association between social support and HRQOL in young adult cancer survivors [44]; however, this study suggests that the impact of family and workplace support varies across physical, mental, and social QOL domains. The findings of this study may help optimize strategies to enhance the HRQOL of young adult cancer survivors. On the other hand, it may be premature to conclude the effectiveness of family and workplace support for HRQOL because our results revealed the small and moderate effect sizes.

In our study, self-reported young adult cancer survivors had lower physical QOL than the general Japanese population aged 20–39 years; but no significant difference was observed in mental QOL. Previous studies have reported that young adult cancer survivors have lower physical and mental QOL than the general population or individuals without a cancer history, even long after treatment completion [12, 13]. Furthermore, previous research suggests that female young adult cancer survivors tend to report lower physical and mental QOL than male survivors [12]. After accounting for age, education, marital status, and race/ethnicity, female young adult cancer survivors showed a greater likelihood of reporting various medical conditions, health-related disabilities, and functional limitations compared to female controls without a cancer history [45]. The lower physical QOL observed in our study’s participants compared to that of the general Japanese population aged 20–39 years was attributed to the large proportion of female participant survivors. However, some studies have reported no difference in mental QOL between young adult cancer survivors and individuals without a cancer history [46], although they had lower mental QOL than individuals without a history of cancer [12, 13]. The lack of a significance difference in mental QOL in this study compared to the general population may be attributed to the fact that most participants were diagnosed less than five years before the study. Additionally, previous studies have specifically evaluated social outcomes, such as poor vocational and educational attainment [9, 10], high financial burden [11], and lack of positive social support [47], and few studies have specifically evaluated young adult cancer survivors’ life in the view of social aspect of HRQOL. Although our study participants were employed, this study found that social QOL is significantly lower among self-reported young adult cancer survivors than in the general Japanese population. Previous studies focused on physical and mental aspects of young adult cancer survivors’ HRQOL and had not been fully studied this social aspect [12, 13, 45, 46]. Our study examined the social QOL among self-reported young adult cancer survivors, focusing on their social lives that could not be assessed through objective indicators such as employment status or school attendance. It may be crucial to examine social QOL among young adult cancer survivors in other countries and regions, and to emphasize the importance of social QOL in support strategies aimed at improving their overall QOL.

This study demonstrated that family support mediates the association of cancer-related symptoms with physical and social QOL among self-reported young adult cancer survivors, though family support did not mediate the association of cancer-related symptoms with mental QOL. Among female cancer survivors, family support contributes to improving overall QOL by alleviating uncertainty about disease recurrence and prognosis, as well as alleviating mental distress [48, 49]. Family support also fosters problem-focused coping and resilience in cancer survivors, enhancing their ability to manage cancer-related symptoms, and ultimately improving physical QOL [14, 48]. Furthermore, family support and open communication among family members have been linked to reduced feelings of isolation and improved social QOL in cancer survivors [50]. Thus, family support is likely to enhance physical and social QOL by mitigating cancer-related uncertainty, fostering problem-focused coping and resilience, and reducing loneliness.

In this study, workplace support mediated the association between cancer-related symptoms and mental and social QOL among self-reported young adult cancer survivors, which support our hypothesis. Workplace support helps alleviate depressive symptoms and mental distress, facilitate adjustment to the cancer experience, and contribute to improved mental QOL [51, 52]. Workplace support may reduce the impact of depression and other cancer-related complications on the mental QOL. AYA cancer survivors engage in work not only to build social connections but also to achieve self-fulfillment and contribute to society [51]. Cancer survivors often experience social isolation due to their illness and treatment leading to feelings of disconnection from everyday life [50]. Workplace support provides social connections, reduces isolation, thereby improving social QOL [53]. By facilitating social participation and its continuation through employment, workplace support fosters self-fulfillment and social contribution, ultimately enhancing their social well-being among cancer survivors [54, 55]. Given that employment represents both a return to normalcy and a form of social contribution for AYA cancer survivors, workplace support that facilitates employment and continuity is likely to positively impact mental and social QOL.

The findings of this study suggest the importance of interventions for family and/or workplace support tailored to impaired aspects of QOL. Interventions for childhood cancer survivors and their families adopted a family-system approach [56, 57]. This approach was based on the Calgary Family Assessment Model, the Calgary Intervention Model, and the Illness Belief Model to enhance family support and reduce illness-related suffering. These interventions first identified family members’ strengths and weaknesses, followed by asking therapeutic questions for family members and creating a supportive environment between family members [56, 57]. A family systems approach could be effective in ensuring young adult cancer survivors receive sufficient support from their families. Furthermore, collaboration between healthcare staff and workplaces is crucial for supporting young adult cancer survivors [58]. Both healthcare professionals at hospitals and workplaces should gather job-related information from survivors and share details regarding diagnosis, treatment, complications, job tasks, and necessary accommodations with employers, supervisors, and occupational health staff. Japan’s Basic Plan to Promote Cancer Control Programs encourages employers to support survivors in balancing their work and treatment [59]. Labor and social security attorneys or social workers may advise on workplace laws and welfare systems, and cancer survivors tend to utilize workplace systems and policies which were specifically designed to support employees with an experience of cancer. Effective communication between healthcare staff and the workplace increases the likelihood of a supportive workplace environment for young adult cancer survivors.

Our study has certain limitations. First, we recruited the study participants through the commercial panel system of Macromill, Inc., and their cancer diagnoses were verified based on self-reports. Although we confirmed whether participants consistently reported their cancer sites and treatment at screening and completing questionnaires, this did not verify the validity of their diagnosis itself. Thus, our findings should be interpreted with caution. Future studies should inspect these findings for young adult cancer survivors who were verified using medical records or recruited through healthcare professionals. Second, our study may include younger adult cancer survivors in better health who were able to complete the questionnaire and work. In our study, 78% of the cancer survivors were women, reflecting the high proportion of young adult cancer survivors diagnosed with breast, uterine, or ovarian cancer in Japan [60]. In addition, three quarters of our study participants reported more than high school graduate, while half of Japanese general population graduated from university or graduate school [61]. Therefore, the generalizability of these findings is limited, and we should extensively recruit young adult cancer survivors through hospitals and patient’s associations in the future. Third, 67% of participants had permanent employment. Temporary workers may be less likely to perceive workplace support owing to the short-term nature of their contracts. Our study may have overestimated the relationship between workplace support and QOL. Fourth, patient groups that provide peer support are still developing in Japan, and few young adult cancer survivors are currently receiving peer support [62]. Therefore, the impact of peer support on HRQOL among young adult cancer survivors should be examined in future studies. Finally, while our SEM model demonstrated good fit, the cross-sectional observational design prevents definitive conclusions about causality between cancer-related symptoms, family and workplace support, and job performance. Further longitudinal studies are required to confirm this causal association.

## Conclusions

Our findings indicate that family support mediates the of the relationship between cancer-related symptoms and physical and social QOL, while workplace support mediates the association of cancer-related symptoms with mental and social QOL. Notably, social QOL among self-reported young adult cancer survivors was significantly lower than that of the general Japanese population aged 20–39 years. Therefore, strategies targeting family and workplace support may be crucial for Japanese young adult cancer survivors to maintain and improve their social QOL. A family system approach and collaboration between healthcare staff and young adult cancer survivors’ workplaces may be effective in enhancing family and workplace support. 

## Data Availability

The datasets generated and/or analyzed during the current study are not publicly available because the authors did not receive approval from the Ethics Committee of the principal investigator’s institution to publicly share the collected data. However, the data are available from the corresponding author upon reasonable request.

## Abbreviations

AGFI: Adjusted Goodness-of-Fit Index
CFI: Comparative Fit Index
CFS: Cancer Fatigue Scale
CMIN/df: Chi-square statistics divided by degrees of freedom
EORTC QLQ-C30: European Organization for Research and Treatment of Cancer Quality of Life Questionnaire Core 30
GFI: Goodness-of-Fit Index
K6: Kessler-6
MCS: Mental Component Summary
MSPSS: Multidimensional Scale of Perceived Social Support
New BJSQ: New Brief Job Stress Questionnaire
PCS: Physical Component Summary
RCS: Role/social Component Summary
RMSEA: Root Mean Square Error of Approximation
SD: Standard Deviation
SEM: Structural Equation Modeling
